# A double dissociation between memory span and word processing among neurological patients attests to the functional independence of verbal short‐term memory

**DOI:** 10.1111/jnp.70013

**Published:** 2025-10-07

**Authors:** Tobias Bormann, Margret Seyboth, Dorothee Kümmerer, Volkmar Glauche, Michel Rijntjes, Cornelius Weiller

**Affiliations:** ^1^ Klinik für Neurologie Und Neurophysiologie Universitätsklinikum Freiburg, Medizinische Fakultät, Albert‐Ludwigs‐Universität Freiburg Freiburg Germany; ^2^ Seminar für Sprachwissenschaft, Universität Erfurt Erfurt Germany; ^3^ Ergotherapie Akademie Südwest Freiburg Freiburg Germany

**Keywords:** aphasia, language, short‐term memory, working memory

## Abstract

Reports of patients with impaired verbal short‐term memory are central to the debate of whether there are independent short‐term stores or whether immediate repetition is supported by activated long‐term memory. Patients with selective impairments of verbal short‐term memory support models with independent buffers. However, it has been argued that these patients were too rare to provide reliable data. Second, it has been suggested that these patients might suffer from subtle impairments of word perception, comprehension or production which previous studies had failed to notice. Ten neurological patients were assessed. Nine participants had impaired immediate spans for digits, letters and words whilst having unimpaired word perception, comprehension and production. Another patient exhibited better preserved immediate repetition despite severely impaired word perception, comprehension and production. This double dissociation provides unequivocal evidence for the functional independence of short‐ and long‐term memory. The size of the present group of STM participants, the largest to date, makes it impossible to ignore data from neuropsychological patients.

## INTRODUCTION

Verbal short‐term memory is the ability to retain a limited number of digits, words, letters, nonwords, or sentences along with their order over a short period of time. This skill is important for coping with everyday life and has received attention from cognitive neuroscience and experimental psychology. In addition, cognitive studies of neuropsychological patients have had a great influence on models of memory and word processing (cf. Shallice, 2019).

For some decades, the focus of cognitive neuropsychology has been on single‐case studies and on dissociations within individual neurological patients, essentially leading to increasingly complex models of cognition. Based on these models, putative functional syndromes have been identified, including the influential short‐term memory (STM) syndrome (Shallice & Papagno, 2019; Shallice & Vallar, 1990), argued to reflect a selective deficit of a verbal short‐term store or buffer. The syndrome is defined as a selective impairment in the recall of sequences of verbal items, words, pseudowords, digits, or letters, which does not result from impaired word perception, comprehension, or production (Shallice & Papagno, 2019; Shallice & Vallar, 1990). Shallice and Papagno (2019) searched the available literature between 1969 and 2015 and found 20 neuropsychological patients all exhibiting a disproportionate deficit of STM capacity despite normal word production. This has been taken to support the notion of a verbal short‐term store functionally independent of word processing (Baddeley, 1990; Norris, 2017). The situation is different when it comes to sentences and discourse. Keeping track of context, integrating words into a coherent discourse model, and resolution of pronouns all have been shown to require verbal working memory (e.g., Almor et al., 1999; Caplan & Waters, 1999).

In recent years, however, there has been a shift towards purportedly simpler cognitive models. In the area of verbal short‐term memory, this approach conceives of short‐term memory as activated parts of long‐term memory (aLTM, Cowan, 2019; Buchsbaum & D'Esposito, 2019). Auditorily presented stimuli are processed phonologically, and the respective representations, lexical entries, and concepts, are activated in a person's mental lexicon and semantic memory. In repetition tasks, these elements are then produced as a result of activation flow from the semantic system to the mental lexicon and subsequent phonological encoding (e.g., Nozari et al., 2010).

This account predicts that short‐term memory performance should be affected by the ability to comprehend and produce single words and, indeed, this has been confirmed in some studies. For example, correlations between phonological and semantic skills and performance on short‐term memory tasks have been reported in individuals with aphasia (e.g., Martin & Ayala, 2004). Repetition of lists of words is better if these consist of items a patient is able to comprehend and reproduce reliably (e.g., Jefferies et al., 2005). In addition, preserved lexical representations have been shown to contribute to repetition of word lists despite impaired semantic comprehension (Papagno et al., 2013). Note, however, that there are ways to accommodate these correlations within models with independent short‐term memory, either by means of additional, domain‐independent buffers (Baddeley & Hitch, 2019) or by means of close interactions of short‐term memory and permanent semantic, lexical, and phonological representations. Therefore, an influence of lexical and semantic variables on repetition can be accommodated within models with independent short‐term buffers (Norris, 2017; Shallice & Papagno, 2019).

Patients with selective impairments of verbal short‐term memory obviously pose a challenge to above‐mentioned models conceiving of short‐term memory as aLTM (e.g., Norris, 2017) and have even been called a “smoking gun argument” against these accounts (cf. Morey et al., 2019). How can the patients' impaired repetition of longer verbal sequences be explained with impaired word perception, comprehension, and production if these have been shown to be intact? Proponents of the above‐mentioned accounts have adopted two lines of arguments to deal with this challenge. First, they claim that since these patients were rare, they may have had atypical language organization or may have other idiosyncratic characteristics which precluded drawing firm inferences from their performance (Buchsbaum & D'Esposito, 2019). Relatedly, Oberauer et al. (2018) have argued that STM patients were, by definition, not a representative sample causing doubts about their replicability and generality.^1^ Second, STM patients may have had subtle deficits in word perception or production which were not adequately assessed (Buchsbaum & D'Esposito, 2019; Morey et al., 2019). The arguments against these cases are reflected in Cowan's (2019, p. 822) claim that “these rare cases can be explained by impairments in encoding, processing, or retrieval related to LTM rather than passive maintenance.” That means subtle impairments in word perception or production have not been noticed by the experimenters and may affect repetition of longer sequences but not single‐word processing.

The present study takes on both of these challenges to cognitive neuropsychology and the idea that vSTM patients are rare and deficits in word processing had simply not been detected. The study contrasts the performance of 10 patients, one suffering from mixed transcortical aphasia (cf. Geschwind et al., 1968) and nine suffering from impaired verbal short‐term memory. All STM patients exhibited repetition deficits without comparable deficits in naming and comprehension of words.

## METHODS

### Participants

Ten neurological patients were included (Table 1). Recruitment involved two strategies: most participants were assessed in the context of a larger project on brain–behaviour relationships (Freiburg Large‐Scale Project). Assessments consisted of a short standardized cognitive test battery in the acute phase within 10 days after the onset of the symptoms. Five participants with a chronic condition were available for more detailed assessments. Their stroke had occurred at a minimum of 2 years before the onset of the present study but up to 19 years (in the case of patient IS). Their performance could be evaluated for the defining qualitative aspects of the ‘classic’ short‐term memory syndrome (cf. Caplan et al., 2012; Vallar et al., 1997): effects of mode of presentation (auditory vs. visual), stimulus length, and stimulus similarity.

**TABLE 1:** Demographic and clinical information on participants.

ID	Sex	Age	YoE	Profession	Handedness	Aetiology	Acute vs. chronic condition	Other neurological deficits
HS	F	68	20	Physician	Right	Haemorrh. (LH)	Acute	
AG	F	59	11	Shopkeeper	Right	Ischaemia (LH)	Acute	
AH	F	42	11	Nurse	Right	Ischaemia (LH)	Acute	
DN	M	78	18	Engineer	Right	Ischaemia (LH); Haemorrh. (LH)	Chronic	Scotoma (RVF), alexia
EK	F	65	18	Economist	Right	Ischaemia (LH)	Chronic	
IS	F	64	11	Secretary	Right	Haemorrh. (RH) + ischaemia (LH)	Chronic	
JB	M	63	18	High school teacher	Right	Ischaemia (LH)	Chronic	
KK	F	79	11	Secretary	Right	Ischaemia (RH) + ischaemia (LH)	Chronic	Hemianopia (LVF)
PB	F	42	13	Kindergarten teacher	Right	T1 Gd‐enhancing lesions LH	Acute	CVID
RJM	M	56	12	Technician	Right	Ischaemia (LH)	Acute	

Abbreviations: CVID, common variable immunodeficiency; LH/RH, left/right hemisphere; RHV/LVF, right/left visual field; YoE, years of education.

Nine participants were included because of severely impaired repetition of sentences and reduced immediate memory spans for digits and other stimuli. One (HS) was included because of better preserved repetition despite impaired single‐word comprehension and production. Four participants (HS; AG; AH; RJM) were assessed in the acute phase after a stroke (haemorrhage; ischaemia) in four sessions of about 15 minutes over the course of two subsequent days. During these sessions, the participants did not report nor exhibit fatigue, and their condition was stable. One participant (PB) suffered from T1 GD‐enhancing lesions in the left inferior parietal region due to Common Variable Immunodeficiency Syndrome (CVID). One participant (DN) had suffered from ischaemia in the year 2000 and from a left‐sided haemorrhage in 2007, causing his current complaints. IS and KK suffered from bilateral CVAs (IS: right‐sided haemorrhage, left‐sided ischaemia; KK: bilateral ischaemias). DN's peripheral alexia and IS's STM deficit have been reported previously (Bormann, Seyboth, et al., 2015; Bormann, Wolfer, et al., 2015). All participants volunteered and gave informed consent. The study was approved by the Internal Review Board (121/12 and 281/13).

**FIGURE 1:**
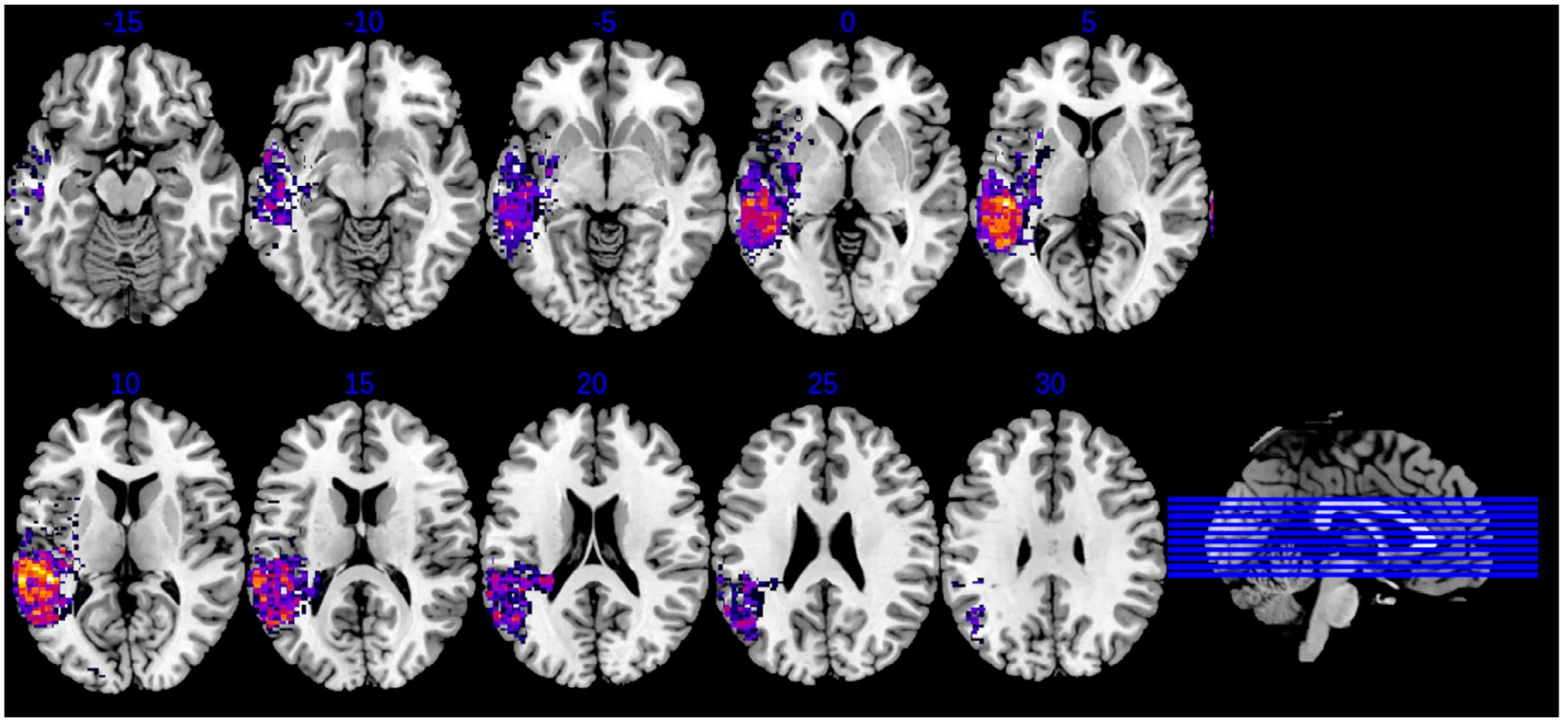
Lesion overlap of participants showing the participants' lesions in the superior temporal and inferior parietal areas of the left hemisphere (left hemisphere corresponds to the left side of the slices).

CT scans were available for two participants (JB; DN), and MRI scans were available for the rest. The largest overlap of lesions was in inferior parietal and superior temporal areas in the left hemisphere (Figure 1).

The participants considered “STM patients” were unimpaired in informal conversations but all scored in the impaired range of the digit span task of the Wechsler Memory Scale‐R when assessed clinically. For this, the percentile rank of digit span was based on the age‐adjusted scores of neurotypical participants provided in the manual of the Wechsler Memory Scale‐R. Seven participants were also assessed with nonverbal short‐term memory (Forward Block Tapping from the Wechsler Memory Scale‐R) where all performed better in comparison to digits. Eight participants were assessed with tests of verbal episodic memory, one being impaired on delayed recognition and the others performing normally (Table 2).

**TABLE 2:** STM patients' memory scores.

ID	Digits trials correct	Digits PR	Blocks trials correct	Blocks PR	Episodic memory test	Delayed recognition correct	Delayed recognition PR
AG	3	<2	7	27	D‐OCS	50%	Impaired
AH	1	<2	5	2	D‐OCS	100%	Unimpaired
DN	1	<2	8	55	CERAD	100%	78
EK	1	<2	5	2	n.a.		
IS	4	2	7	25	CERAD	100%	65
JB	4	5	6	8	CERAD	100%	71
KK	3	<2	7	30	D‐OCS	75%	Unimpaired
PB	3	<2	n.a.		HVLT‐R	100%	79
RJM	3	<2	n.a.		CERAD	100%	77

Abbreviations: CERAD, Consortium to Establish a Registry for Alzheimer's Disease test battery; D‐OCS, Oxford Cognitive Screen (German Version); HVLT‐R, Hopkins Verbal Learning Test revised; n.a., not administered; PR, percentile rank.

### Materials and analyses

Word processing was assessed with four subtests of the neurolinguistic battery “Lexikon Modellorientiert” (LeMo, De Bleser et al., 2004): oral naming, auditory lexical decision, word‐picture matching, and word repetition. For all subtests, a normal range is defined based on extensive evaluation with healthy participants.

Oral naming consisted of 20 one‐syllable target words of low (*n* = 10) and high frequency (*n* = 10). All target words were concrete nouns. The same nouns are used for a word‐picture matching task with the target picture and three distractors. Two distractors are semantically related, and one is unrelated. Naming and word‐picture matching were not carried out during the same session. Auditory lexical decision involved 40 one‐syllable nouns varying in frequency and concreteness. Word trials were mixed with the same amount of nonword trials. Letters in the target words were changed to generate a pronounceable and phonotactically legal nonword. Word repetition involved the same 40 nouns. The first response was scored. If no response occurred within 15 s of the naming trial, the response was scored as an omission.

Span tasks involved digit span (length 2 to 7 items, 10 lists for each length; total 60 lists), phonologically dissimilar letters (length 2 to 6 items, 10 lists for each length; total 50 lists), and phonologically dissimilar one‐syllable words (length 2 to 5 items, 10 lists for each length; total 40 lists). Sequences were read to the individual participants at a rate of one item per second; sequences were scored as correct if the items were reproduced in the correct order.

For the word processing task, the individual scores were judged as normal or impaired based on the available range of normal performance. Comparisons between patients were based on Crawford et al.'s (2011) modified *t*‐test using the available standard deviation from 41 healthy controls as provided in the test manual. For the three different span tasks, the individual patient's score was compared to the mean of a group of 20 elderly unimpaired control participants using Crawford and Garthwaite's (2002) modified *t*‐test. Differences between participants were assessed with Crawford et al.'s (2011) *t*‐test using the standard deviation from the controls.

For the repetition tasks, the position of the errors within the sequences was scored using the normalization algorithm of Machtynger and Shallice (2009, p. 222). This method allows the calculation of error positions for sequences of different lengths. In the present case, errors in sequences of 3, 4, and 5 items of length were normalized to five regions, and errors in these regions were summed for the participants.

For five STM patients, the influence of word length, phonological similarity, and visual versus auditory presentation was assessed as it has been argued that in STM patients, the robust effects of similarity, length and modality are missing (e.g., Baddeley, 1990; Vallar et al., 1997). Similar versus dissimilar words were matched for length, frequency, concreteness, and part of speech. Three‐ and one‐syllable words were also matched on all relevant variables. For the effect of modality, digits were presented on a screen or auditorily at a speed of one item per second.

### Control participants

For the word processing tasks (Naming, Lexical decision, Word‐picture matching, Word repetition), 41 healthy individuals volunteered. Their mean age was 59.7 years. They had normal hearing and vision and German as their first language (De Bleser et al., 2004). For the repetition tasks, 20 healthy controls were assessed. They had normal hearing and vision and were between 51 and 78 years old (mean age 59.1 years). German was their first language.

## RESULTS

Table 3 provides the results of participants on four tests of word processing. All participants were unimpaired on the word repetition task. HS was severely impaired on the two‐word perception and comprehension tasks and on the naming task. In contrast, all STM patients performed in the normal range on these three word tasks and were formally unimpaired. Using the standard deviation from the manual of LeMo, a significantly better performance was observed for all STM patients on the naming task: In comparison to HS' score (10 out of 20), a score of 18 (participants DN and EK) was significantly better (*t* = 11.3, *p* < .01). The other STM patients performed even better. In the lexical decision task, HS (64 out of 80) performed significantly lower than AH (75 out of 80) (*t* = 9.7, *p* < .01) and all other STM patients. In the word comprehension task, HS' score was significantly lower than that of the STM participants (14 vs. 19 correct, *t* = 8.8, *p* < .01).

**TABLE 3:** Tests of single‐word processing.

ID	Naming	Repetition	Lexical decision	Word‐picture match.
Maximum score	20	40	80	20
Normal range	19–20	37–40	73–80	19–20
Ctrls' stddev	.5	n.a.	.8	.4
HS	10 (50%)	40 (100%)	64 (80%)	14 (60%)
AG	20 (100%)	40 (100%)	76 (95%)	20 (100%)
AH	19 (95%)	40 (100%)	75 (94%)	20 (100%)
DN	18 (90%)	40 (100%)	79 (99%)	20 (100%)
EK	18 (90%)	40 (100%)	79 (99%)	19 (95%)
IS	19 (95%)	40 (100%)	78 (98%)	19 (95%)
JB	19 (95%)	40 (100%)	79 (99%)	20 (100%)
KK	19 (95%)	38 (95%)	76 (95%)	19 (95%)
PB	20 (100%)	40 (100%)	80 (100%)	20 (100%)
RJM	19 (95%)	n.a.	79 (99%)	20 (100%)

Abbreviation: n.a., not administered.

Table 4 provides the mean and standard deviation of the control group and the scores of all participants on the span tasks. All 10 patients had impaired digit span, meaning that a score of 39 correct lists (HS) was significantly impaired (*t* = −3.5, *p* < .01). All patients were impaired on the letter span and word span tasks. HS's score of 34 lists of letters was significantly impaired in comparison to the controls' mean and standard deviation (*t* = −2.1, *p* < .05). PB, the best performing STM patient's score of 30 correct lists, was significantly impaired (*t* = −2.7, *p* < .02). Significantly impaired performance was also observed for all patients for word list repetition (best performing participant PB: *t* = −4.8, *p* < .01).

**TABLE 4:** Span task performance.

	Digits	Letters	Words
Maximum score	60	50	40
Ctrls' mean (SD)	55.0 (4.4)	45.9 (5.6)	37.2 (2.5)
HS	39	34	20
AG	27	n.a.	23
AH	25	20	15
DN	17	11	9
EK	27	17	15
IS	24	17	16
JB	23	n.a.	12
KK	18	10	6
PB	35	30	25
RJM	23	22	14
STM pts. mean (SD)	24.0 (5.6)	18.1 (6.8)	14.0 (5.6)

Abbreviation: n.a., not administered.

When comparing the STM patients to HS on the digit span task, PB was not different (35 vs. 39 correct, *t* < 1.0). The difference for AG and EK (27 correct) vs. HS missed significance on a two‐sided test (*t* = 1.9, *p* < .07). AH's and the other STM patients' performance was significantly lower (*t* = 2.3, *p* < .05). With letters, PB was numerically poorer than HS but not significantly. Those STM patients (DN, EK, IS, KK) who achieved 17 or fewer correct lists were all significantly impaired in comparison to HS (*t* = 2.1, *p* < .05). Finally, the differences for lists of words were smaller, but DN, JB, and KK performed significantly lower than HS (JB: 12 lists correct; HS: 20 correct; *t* = 2.3, *p* < .05). The difference between HS on the one hand and KK and DN on the other was highly significant (*t* = 3.1 and 3.9, *p* < .01). When compared to the STM patients as a group, HS was significantly better on digits (*t* = 2.5, *p* < .05). Her advantage for letters missed significance (*t* = 2.2, *p* < .06).

**FIGURE 2:**
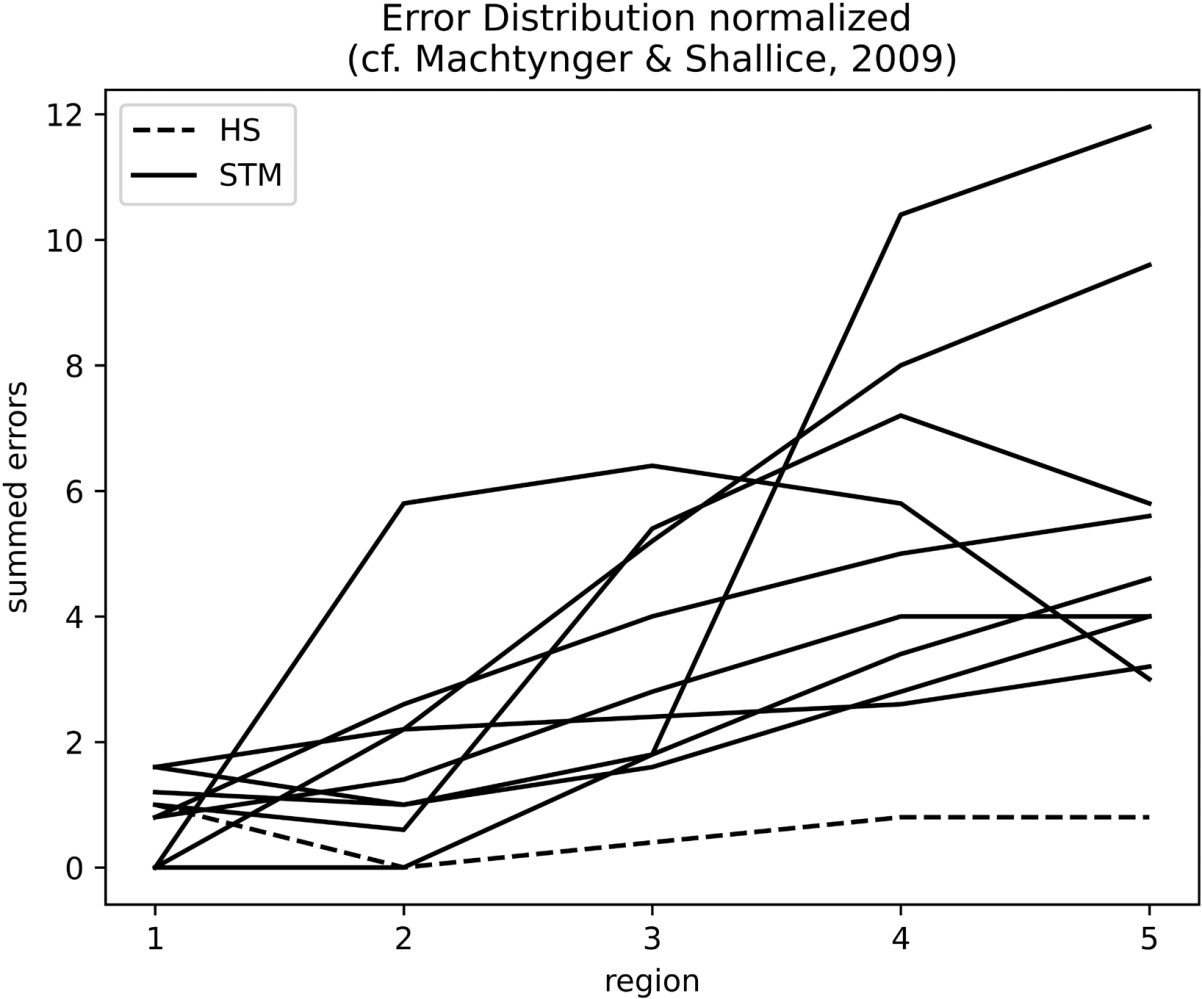
Serial position curves.

The distribution of errors across the sequences of digits is plotted in Figure 2 for all participants. HS produced fewer errors than the STM patients, and her serial position curve was flat. In contrast, the STM patients exhibited an increase in errors across the sequence. Two participants with impaired short‐term memory (PB, EK) produced fewer errors in the final position.

The effects of length, phonological similarity and mode of presentation are provided in Table 5. The controls all performed better with dissimilar words and slightly better with shorter words. The younger controls (between 50 and 65 years) exhibited an advantage for auditory over visual presentation. This effect was less obvious in an older group of controls (75–80 years old). The majority of STM patients performed better with visually presented digits and exhibited a smaller or absent similarity effect.

**TABLE 5:** Modality and length effects on span in a subgroup of STM patients.

ID	Age	Auditory digit span	Visual digit span	Word span dissimilar 1‐syllable	Word span similar 1‐syllable	Word span 3‐syllables
Older ctrls' mean (*n* = 5)	77.6	42.4	50.2	34.0	24.8	31.6
DN	78	15	23	5	6	2
Younger ctrls' mean (*n* = 10)	56.7	55.1	47.9	39.2	29.1	35.3
EK	65	27	27	15	16	11
IS	64	24	29	15	15	8
JB	63	23	20	12		10
PB	42	31		25	18	22

## DISCUSSION

Patients with impaired verbal short‐term memory (vSTM) have been a critical piece of evidence for models of short‐term and working memory. Selective impairments of vSTM despite preserved language processing and other memory functions suggest the existence of selective and independent mechanisms to represent a limited set of items along with their order. They are, thus, compatible with multi‐component models of verbal short‐term memory and have sometimes even been referred to as a “smoking gun” argument for the validity of these models (cf. Morey et al., 2019; Norris, 2017; Shallice & Papagno, 2019). Proponents of alternative models, conceiving of working memory as activated long‐term memory, have either argued that STM patients were too rare and posed exceptions with idiosyncratic characteristics or that deficits in word processing have been missed and so‐called “pure STM patients” weren't pure at all. Other authors doubt patient studies' replicability and generalizability (Oberauer et al., 2018).

The present study, first, reports the largest group of patients with selective impairment of verbal short‐term memory to date. All nine STM patients exhibited formally unimpaired picture naming, auditory perception, word comprehension, and word repetition. In contrast, they were all impaired on three different verbal short‐term memory tasks. They thus exhibit a selective verbal short‐term memory impairment. Lesions were compatible with previous reports (Baldo et al., 2012; Leff et al., 2009). They all matched the criteria proposed by Shallice and Papagno (2019). Learning of new word forms has been shown to be impaired for virtually all individuals with impaired verbal short‐term memory (e.g., Page & Norris, 2009). This was formally assessed in two of our participants, revealing severely impaired new word learning. Learning of one of these two participants was reported previously (Bormann, Seyboth, et al., 2015; Bormann, Wolfer, et al., 2015).

In addition, results from these participants are contrasted with another individual, HS, with severely impaired speech perception, word comprehension, and lexical access, yet better preserved STM capacity. This latter condition is known as mixed‐transcortical aphasia (Geschwind et al., 1968). Comprehension of words involves phonological processing, accessing a word in one's mental lexicon, and, subsequently, accessing its meaning in semantic memory. In spontaneous speech and picture naming, a concept in semantic memory activates its corresponding entry in the lexicon (a word form, sometimes preceded by activating a lemma) which is then encoded phonologically and articulated. In HS, phonological processing of auditory input is impaired as well as accessing the lexicon from the semantic system. This leads to impaired auditory comprehension as well as impaired object naming. In those models of short‐term memory which conceive of short‐term memory as activated long‐term memory, HS' deficits should affect her ability to repeat words and other stimuli.

The different analyses yielded significant differences among these patients. The STM patients were all better than HS at single‐word processing. In contrast, most of them performed poorly when repeating lists of digits and letters. This pattern emerged in comparison of individual participants but also when the STM patients were treated as a group.

Two conclusions can be drawn from the present study. First, the condition known as “pure short‐term memory” is not as rare as claimed by non‐clinicians. Shallice and Papagno (2019) summarised 20 reports published between 1969 and 2015, including one of our participants, IS. The present study now adds eight further cases, an increase of 40%.^2^ The sheer size of our sample makes it difficult to dismiss these neuropsychological cases as irrelevant and makes it less easy to argue for atypical language organisation. All STM patients here were right‐handed, all had undergone normal school and a range of professional training, including university. There is nothing to suggest that any of our participants had atypical language organisation. In addition, some were assessed in the acute phase after their stroke while others were in the chronic phase.

Second and more importantly, the STM patients and participant HS form a double dissociation between single‐word processing and verbal short‐term memory. The main result of the comparisons is significantly better processing and comprehension of auditory words and the production of words in response to pictures in the STM patients. Of the group of nine participants with impaired vSTM, two may be considered not entirely pure cases: DN had normal spontaneous speech and word production yet tended to produce phonemic paraphasias when repeating longer sentences. JB's spontaneous speech was slightly nonfluent and effortful. None of these two participants produced any phonemic paraphasias in digit span tasks, though. Phonological impairments were so mild as not to impair the production of sequences of three words. Thus, word perception, comprehension, and production are better preserved in nine participants. In contrast, they all suffer from impaired verbal short‐term memory, documented by selective impairments in the repetition of digits, words, and letters. The performance of HS, in contrast, shows the opposite pattern and completes a double dissociation. HS was impaired in processing, comprehension, and production of single words while at the same time being better able to repeat sequences of digits, words, and letters. If deficits in auditory perception, word comprehension, or production were the underlying cause of impaired verbal short‐term memory, as claimed by some authors (Cowan, 2019), HS should have performed worse than the STM patients. Clearly, this was not the case, and the claim that subtle deficits in speech perception or language production underlie impaired vSTM (Buchsbaum & D'Esposito, 2019; Cowan, 2019; Morey et al., 2019) is rendered invalid.

The distribution of errors also differed between HS and the STM patients. The STM patients exhibited no error in the first positions of the presented sequences and an increase towards the end. In contrast, HS' error curve was flat. Not all STM patients could be assessed for the effects of phonological similarity, word length, and visual versus auditory presentation, but a qualitative difference could be observed for most participants.

All participants in the present study were better with digits than letters and performed worst with words. While some authors (Morey et al., 2019) claimed this to be at odds with a purely auditory retention mechanisms, this might reflect familiarity with different types of sequences (Jones & Macken, 2015). Alternatively, digits constitute a smaller set to choose from than words, which might make it easier to reproduce the sequences. Both these explanations, thus, assume a role of prior experience, i.e. long‐term memory, on immediate repetition. In addition, influences of long‐term memory on immediate retention can be accommodated into models with independent buffers (Shallice & Papagno, 2019).

The present results rule out deficits in primary word perception and production as the reason for impaired vSTM, but they do not unambiguously support a specific model of short‐term memory. The results are compatible with the multi‐component model of working memory (Baddeley & Hitch, 2019), but may also be compatible with other accounts. For example, the STM patients could suffer from impaired mapping of the auditory input to motor output, as suggested by Buchsbaum and D'Esposito (2019). In this model, a repetition deficit could result from impaired input–output mapping, with primary auditory word processing and production spared. This, however, fails to account for the pattern exhibited by HS: her impaired speech perception and production would inevitably affect repetition, despite preserved input–output mapping. It remains to be seen whether STM patients can be simulated in dual‐route computational models of word repetition (e.g., Nozari et al., 2010), as suggested by Buchsbaum and D'Esposito (2019).

Other models of working memory have proposed amodal mechanisms to maintain the order of items in working memory (Majerus, 2013) or an amodal focus of attention (Cowan, 2019; Cowan et al., 2021). Damage to these mechanisms may cause the short‐term memory syndrome. Retention of nonverbal sequences was not systematically evaluated in our participants, but at least three of our participants were assessed with the Corsi block tapping test. Of these three, two were found to perform normally (AG: percentile rank [PR] above 40; IS: PR above 25) and one was even above average (DN: PR >90). A more systematic evaluation of the retention of verbal and nonverbal sequences is underway. However, in the models of Majerus (2013) and Cowan (2019), impaired processing of words should inevitably impair the retention of verbal information because items held in short‐term memory require efficient lexical and semantic processing. These models are, thus, difficult to reconcile with the double dissociation found here.

To conclude, the present study reports nine patients with selectively impaired verbal short‐term memory despite preserved single‐word comprehension and production. Another patient presented with the reverse dissociation, namely better preserved digit, letter, and word spans despite severely impaired single‐word processing. This double dissociation invalidates explanations of impaired vSTM as resulting from subtle, previously undetected impaired single‐word processing. In addition, the argument that patients with pure STM deficits are rare and may not inform cognitive models of memory cannot be upheld.

## AUTHOR CONTRIBUTIONS

**Tobias Bormann:** Conceptualization; investigation; writing – original draft; methodology; validation; visualization; writing – review and editing; software; formal analysis; data curation; project administration. **Margret Seyboth:** Investigation; conceptualization; writing – review and editing; methodology; validation; data curation. **Dorothee Kümmerer:** Investigation; writing – review and editing; data curation; validation. **Volkmar Glauche:** Conceptualization; writing – review and editing; visualization; methodology; software; formal analysis; data curation. **Michel Rijntjes:** Conceptualization; writing – review and editing; investigation; methodology; supervision. **Cornelius Weiller:** Conceptualization; writing – review and editing; data curation; supervision; project administration.

## CONFLICT OF INTEREST STATEMENT

The authors declare no conflict of interest to declare.

## Data Availability

The data that support the findings of this study are available on request from the corresponding author. The data are not publicly available due to privacy or ethical restrictions.

## Footnotes

^1^
In contrast, according to Norris (2017, p. 993), the “primary data” in favour of models with separate short‐term and long‐term memory “come from studies of STM patients”.

^2^
At least five more German‐speaking participants are known to the authors who, however, could not be included in the present study. Two patients were reported in a German manuscript by Dittmann (1996), three others could not be contacted after they had been seen as outpatients.

## References

[jnp70013-bib-0001] Almor, A. , Kempler, D. , MacDonald, M. C. , Andersen, E. S. , & Tyler, L. K. (1999). Why do Alzheimer patients have difficulty with pronouns? Working memory, semantics, and reference in comprehension and production in Alzheimer's disease. Brain and Language, 67, 202–227. 10210631 10.1006/brln.1999.2055

[jnp70013-bib-0002] Baddeley, A. D. (1990). The development of the concept of working memory: Implications and contributions of neuropsychology. In G. Vallar & T. Shallice (Eds.), Neuropsychological impairments of short‐term memory (pp. 54–73). Cambridge University Press.

[jnp70013-bib-0003] Baddeley, A. D. , & Hitch, G. J. (2019). The phonological loop as a buffer store: An update. Cortex, 112, 91–106. 29941299 10.1016/j.cortex.2018.05.015

[jnp70013-bib-0004] Baldo, J. V. , Katseff, S. , & Dronkers, N. F. (2012). Brain regions underlying repetition and auditory‐verbal short‐term memory deficits in aphasia: Evidence from voxel‐based lesion symptom mapping. Aphasiology, 26(354), 338. 24976669 10.1080/02687038.2011.602391 PMC4070523

[jnp70013-bib-0005] Bormann, T. , Seyboth, M. , Umarova, R. , & Weiller, C. (2015). “I know your name, but not your number” – Patients with verbal short‐term memory deficits are impaired in learning sequences of digits. Neuropsychologia, 72, 80–86. 25823999 10.1016/j.neuropsychologia.2015.03.027

[jnp70013-bib-0006] Bormann, T. , Wolfer, S. , Hachmann, W. , Neubauer, C. , & Konieczny, L. (2015). Fast reading responses in pure alexia: “Fast, yet serial”. Neurocase, 21(2), 251–267. 24592898 10.1080/13554794.2014.890732

[jnp70013-bib-0007] Buchsbaum, B. R. , & D'Esposito, M. (2019). A sensorimotor view of verbal working memory. Cortex, 112, 134–148. 30639088 10.1016/j.cortex.2018.11.010

[jnp70013-bib-0008] Caplan, D. , Waters, G. , & Howard, D. (2012). Slave systems in verbal short‐term memory. Aphasiology, 26(3/4), 279–316. 10.1080/02687038.2011.642795 PMC3859463 24347786

[jnp70013-bib-0009] Caplan, D. , & Waters, G. S. (1999). Verbal working memory and sentence comprehension. Behavioral and Brain Sciences, 22(1), 77–94. 11301522 10.1017/s0140525x99001788

[jnp70013-bib-0010] Cowan, N. (2019). Short‐term memory based on activated long‐term memory: A review in response to Norris (2017). Psychological Bulletin, 145(8), 822–847. 31328941 10.1037/bul0000199 PMC6650160

[jnp70013-bib-0011] Cowan, N. , Morey, C. C. , & Naveh‐Benjamin, M. (2021). An embedded processes approach to working memory: How is it distinct from other approaches and to what ends. In R. Logie , V. Camos , & N. Cowan (Eds.), Working memory: State of the science (pp. 44–84). Oxford University Press.

[jnp70013-bib-0012] Crawford, J. R. , & Garthwaite, P. H. (2002). Investigation of the single case in neuropsychology: Confidence limits on the abnormality of test scores and test score differences. Neuropsychologia, 40, 1196–1208. 11931923 10.1016/s0028-3932(01)00224-x

[jnp70013-bib-0013] Crawford, J. R. , Garthwaite, P. H. , & Wood, L. T. (2011). Inferential methods for comparing two single cases. Cognitive Neuropsychology, 27, 377–400. 10.1080/02643294.2011.559158 21718213

[jnp70013-bib-0014] De Bleser, R. , Cholewa, J. , Stadie, N. , & Tabatabaie, S. (2004). LEMO‐Lexikon modellorientiert: Einzelfalldiagnostik bei Aphasie, Dyslexie und Dysgraphie; Diagnostikband Sprachverständnis. Urban & Fischer.

[jnp70013-bib-0015] Dittmann, J. (1996). Die exemplarische Analyse eines Arbeitsgedächtnis‐Defizits. Arbeitsberichte der Forschungsgruppe Neurolinguistik am Deutschen Seminar I der Universität Freiburg.

[jnp70013-bib-0016] Geschwind, N. , Quadfasel, F. A. , & Segarra, J. (1968). Isolation of the speech area. Neuropsychologia, 6(4), 327–340.

[jnp70013-bib-0017] Jefferies, E. , Jones, R. W. , Bateman, D. , & Lambon Ralph, M. A. (2005). A semantic contribution to nonword recall? Evidence for intact phonological processes in semantic dementia. Cognitive Neuropsychology, 22(2), 183–212. 21038246 10.1080/02643290442000068

[jnp70013-bib-0018] Jones, G. , & Macken, B. (2015). Questioning short‐term memory and its measurement: Why digit span measures long‐term associative learning. Cognition, 144, 1–13. 26209910 10.1016/j.cognition.2015.07.009

[jnp70013-bib-0019] Leff, A. P. , Schofield, T. M. , Crinion, J. T. , Seghier, M. L. , Grogan, A. , Green, D. W. , & Price, C. J. (2009). The left superior temporal gyrus is a shared substrate for auditory short‐term memory and speech comprehension: Evidence from 210 patients with stroke. Brain, 132(12), 3401–3410. 19892765 10.1093/brain/awp273 PMC2792373

[jnp70013-bib-0020] Machtynger, J. , & Shallice, T. (2009). Normalizing serial position analyses: The proportional accountability algorithm. Cognitive Neuropsychology, 26(2), 217–222. 19347701 10.1080/02643290902820105

[jnp70013-bib-0021] Majerus, S. (2013). Language repetition and short‐term memory: An integrative framework. Frontiers in Human Neuroscience, 7, 357. 23874280 10.3389/fnhum.2013.00357 PMC3709421

[jnp70013-bib-0022] Martin, N. , & Ayala, J. (2004). Measurements of auditory‐verbal STM span in aphasia: Effects of item, task, and lexical impairment. Brain and Language, 89(3), 464–483. 15120538 10.1016/j.bandl.2003.12.004

[jnp70013-bib-0023] Morey, C. C. , Rhodes, S. , & Cowan, N. (2019). Sensory‐motor integration and brain lesions: Progress toward explaining domain‐specific phenomena within domain‐general working memory. Cortex, 112, 149–161. 30612701 10.1016/j.cortex.2018.11.030

[jnp70013-bib-0024] Norris, D. (2017). Short‐term memory and long‐term memory are still different. Psychological Bulletin, 143(9), 992–1009. 28530428 10.1037/bul0000108 PMC5578362

[jnp70013-bib-0025] Nozari, N. , Kittredge, A. , Dell, G. S. , & Schwartz, M. F. (2010). Naming and repetition in aphasia: Steps, routes, and frequency effects. Journal of Memory and Language, 63, 541–559. 21076661 10.1016/j.jml.2010.08.001 PMC2976549

[jnp70013-bib-0026] Oberauer, K. , Lewandowsky, S. , Awh, E. , Brown, G. D. , Conway, A. , Cowan, N. , & Ward, G. (2018). Benchmarks provide common ground for model development: Reply to Logie (2018) and Vandierendonck (2018). Psychological Bulletin, 144(9), 972–977. 30148382 10.1037/bul0000165

[jnp70013-bib-0027] Page, M. P. A. , & Norris, D. G. (2009). A model linking immediate serial recall, the Hebb repetition effect and the learning of phonological word forms. Philosophical Transactions of the Royal Society, B: Biological Sciences, 364, 3737–3753. 10.1098/rstb.2009.0173 PMC2846317 19933143

[jnp70013-bib-0028] Papagno, C. , Vernice, M. , & Cecchetto, C. (2013). Phonology without semantics? Good enough for verbal short‐term memory. Evidence from a patient with semantic dementia. Cortex, 49(3), 626–636. 22664140 10.1016/j.cortex.2012.04.015

[jnp70013-bib-0029] Shallice, T. (2019). The single case study of memory. In S. E. MacPherson & S. Della Sala (Eds.), Cases of amnesia. Routledge.

[jnp70013-bib-0030] Shallice, T. , & Papagno, C. (2019). Impairments of auditory‐verbal short‐term memory: Do selective deficits of the input phonological buffers exist? Cortex, 112, 107–122. 30414628 10.1016/j.cortex.2018.10.004

[jnp70013-bib-0031] Shallice, T. , & Vallar, G. (1990). The impairment of auditory‐verbal short‐term storage. In G. Vallar & T. Shallice (Eds.), Neuropsychological impairments of short‐term memory (pp. 11–53). Cambridge University Press.

[jnp70013-bib-0032] Vallar, G. , Di Betta, A. M. , & Silveri, M. C. (1997). The phonological short‐term store‐rehearsal system: Patterns of impairment and neural correlates. Neuropsychologia, 35, 795–812. 9204486 10.1016/s0028-3932(96)00127-3

